# Models for malaria control optimization—a systematic review

**DOI:** 10.1186/s12936-024-05118-3

**Published:** 2024-10-03

**Authors:** Randolph Ngwafor, Sunil Pokharel, Ricardo Aguas, Lisa White, Rima Shretta

**Affiliations:** 1 Nuffield Department of Medicine, University of Oxford, Oxford, United Kingdom; 2 Department of Biology, University of Oxford, Oxford, United Kingdom

**Keywords:** Optimization, Malaria, Resource allocation, Limited resource setting

## Abstract

**Background:**

Despite advances made in curbing the global malaria burden since the 2000s, progress has stalled, in part due to a plateauing of the financing available to implement needed interventions. In 2020, approximately 3.3 billion USD was invested globally for malaria interventions, falling short of the targeted 6.8 billion USD set by the GTS, increasing the financial gap between desirable and actual investment. Models for malaria control optimization are used to disentangle the most efficient interventions or packages of interventions for inherently constrained budgets. This systematic review aimed to identify and characterise models for malaria control optimization for resource allocation in limited resource settings and assess their strengths and limitations.

**Methods:**

Following the Prospective Register of Systematic Reviews and Preferred reporting Items for Systematic Reviews and Meta-Analysis guidelines, a comprehensive search across PubMed and Embase databases was performed of peer-reviewed literature published from inception until June 2024. The following keywords were used: optimization model; malaria; control interventions; elimination interventions. Editorials, commentaries, opinion papers, conference abstracts, media reports, letters, bulletins, pre-prints, grey literature, non-English language studies, systematic reviews and meta-analyses were excluded from the search.

**Results:**

The search yielded 2950 records, of which 15 met the inclusion criteria. The studies were carried out mainly in countries in Africa (53.3%), such as Ghana, Nigeria, Tanzania, Uganda, and countries in Asia (26.7%), such as Thailand and Myanmar. The most used interventions for analyses were insecticide-treated bed nets (93.3%), IRS (80.0%), Seasonal Malaria Chemoprevention (33.3%) and Case management (33.3%). The methods used for estimating health benefits were compartmental models (40.0%), individual-based models (40.0%), static models (13.0%) and linear regression model (7%). Data used in the analysis were validated country-specific data (60.0%) or non-country-specific data (40.0%) and were analysed at national only (40.0%), national and subnational levels (46.7%), or subnational only levels (13.3%).

**Conclusion:**

This review identified available optimization models for malaria resource allocation. The findings highlighted the need for country-specific analysis for malaria control optimization, the use of country-specific epidemiological and cost data in performing modelling analyses, performing cost sensitivity analyses and defining the perspective for the analysis, with an emphasis on subnational tailoring for data collection and analysis for more accurate and good quality results. It is critical that the future modelling efforts account for fairness and target at risk malaria populations that are hard-to-reach to maximize impact.

*Trial registration*: PROSPERO Registration number: CRD42023436966

**Supplementary Information:**

The online version contains supplementary material available at 10.1186/s12936-024-05118-3.

## Background

Malaria persists as a global health challenge, particularly in low- and middle-income countries (LMICs). The burden of malaria is concentrated in sub-Saharan Africa, contributing to over 95% of global cases [1]. Ten African countries were labelled as "High Burden to High Impact" (HBHI) in 2017 due to their substantial contribution to the global burden [1, 2]. Despite progress in reducing the disease burden from 81 cases per 1000 population at risk in 2000 to 59 cases in 2015, advancements have stalled following the plateauing of deployed resources [1].

The World Health Organization (WHO) has set ambitious targets outlined in the Global Technical Strategy (GTS), aiming to reduce malaria cases and deaths by at least 75% by 2025 and 90% by 2030, compared to 2015 [3]. To meet these targets, the WHO recommends: Prevention, involving interventions such as mass distribution of insecticide-treated bed nets (ITNs), indoor residual spraying (IRS), larviciding, intermittent preventive treatment in pregnancy (IPTp) and infants (IPTi, now PMC), seasonal malaria chemoprevention (SMC); as well as case management, focusing on the diagnosis and treatment of malaria cases at the health facility and community levels [3–5]. Countries through their national control or elimination programmes try to align with these global strategies through country-specific national strategic plans, adopting and implementing country-specific interventions at the national and subnational levels [6, 7].

Scaling up malaria interventions to achieve GTS targets necessitates significant financial support globally and domestically. In 2020, approximately 3.3 billion USD were invested globally for malaria interventions [1], falling short of the targeted 6.8 billion USD set by the GTS, increasing the financial gap between desirable and actual investment [1, 3]. The financial gap poses a significant risk of resurgence, potentially leading to billions of avertable malaria cases and deaths, and costing over 5 billion USD to health systems and communities by 2030 [8]. In 2020, although majority of the global investment for malaria came from international donors, about 33% of investments within countries came from domestic (government) funding [1]. These financial constraints are felt most in LMICs, and a lack of sufficient evidence on country-specific financial costs and effects of different interventions makes it difficult to determine the true efficiency of these interventions [9]. Also, in order to ensure epidemiological and economic efficiency, subnational tailoring needs to be taken into consideration to improve efficiency of interventions [10, 11]. The most efficient interventions or packages of interventions refer to strategies or combinations of strategies that are chosen based on careful analysis of costs, benefits, and contextual factors to achieve the maximum possible reduction in malaria burden with the resources available [12]. It would, therefore, be imperative that models for malaria control optimization are used to disentangle the most efficient interventions or packages of interventions for inherently constrained budget(s). Disease-specific models for optimization have been systematically reviewed for other diseases such as HIV/AIDS [13], but not for malaria, showing the need to understand what models for malaria control optimization are available in the literature.

Mathematical models in malaria research serve several key purposes and are extensively employed to simulate and understand the transmission dynamics of the disease [14]; by simulating the effects of various interventions to assess their impact and inform strategic planning for control and elimination efforts [15], for example in evaluating the cost-effectiveness of different strategies and identifying the most promising combinations of interventions [16]. One significant application of mathematical modelling in malaria is the optimization of intervention strategies. Optimization in the context of malaria modelling refers to the use of mathematical techniques to identify the best possible strategies or determine the optimal mix and coverage levels of interventions or the geographic targeting of resources for achieving specific objectives, such as reducing malaria transmission or minimizing costs [10, 16–18]. The optimization process typically involves defining the following: an objective function that represents the goal of the optimization such as to minimize the number of malaria cases or deaths; constraints such as a budget constraint which are the limitations or restrictions considered in the optimization process; and optimization techniques such as linear programming, or integer programming that are used to solve the optimization problem given the objective function and constraints [13, 19]. While mathematical models provide valuable insights and theoretical optimization solutions, implementing these solutions in the real world requires consideration of several practical factors:

(i) The implementation of optimized malaria strategies often needs to align with national health policies, priorities, and political realities. For instance, there might be political resistance to certain interventions or a preference for locally developed strategies over those recommended by external modelling efforts [2].
(ii) Optimized strategies derived from models must also be feasible from a logistical standpoint. This includes the availability of resources, infrastructure, and personnel to carry out the interventions effectively. Real-world constraints such as supply chain issues, geographical barriers, and weather conditions can significantly affect the feasibility and effectiveness of optimized plans [20, 21].
(iii) Community acceptance and adherence to interventions are crucial for their success. Optimized strategies must consider local cultural practices, beliefs, and social dynamics that could influence the uptake of interventions like ITNs or IRS [22, 23].
(iv) Real-world conditions are often dynamic and unpredictable. Optimization strategies need to be flexible and adaptable to changing conditions, such as shifts in malaria transmission patterns due to climate change or evolving resistance to anti-malarial drugs [24].

To optimize resource allocation for malaria control, various mathematical models have been employed in combination with economic/cost models. These include individual-based mathematical models [25–27], a geospatial dynamic transmission epidemic model [16] and compartmental transmission models [28, 29]. Although one study in Senegal demonstrated the impact of improving allocative efficiency to scale up malaria intervention packages, no model was used. Rather, they aggregated annual cost estimates of the considered intervention packages to provide data for better programmatic decision-making [30]. While some studies implemented their models across multiple countries in Africa [25, 26] and Asia [29], others were country specific [16, 28]. Within the latter subset, some performed subnational data analysis, emphasising subnational tailoring in order to account for context-specific drivers of intervention efficacy and thus achieving more precise results.

A systematic literature review is presented here, to identify available models for malaria control optimization for resource allocation in limited resource settings, determine how they have been used and to assess them for quality and utility.

The research question for this review was “Are there English-language publications in peer-reviewed scientific journals describing how models have been used to allocate resources for malaria interventions in limited resource settings?” The main aim of this review was to identify and characterize models for malaria control optimization for resource allocation in malaria control and elimination settings and to identify their strengths and limitations.

### Methods

A systematic search of peer-reviewed literature published from inception until June 2024 was conducted. The Preferred reporting Items for Systematic Reviews and Meta-Analysis protocols (PRISMA) 2020 guidelines were used to report the findings (Additional file 1). The protocol for the systematic review was registered with the international Prospective Register of Systematic Reviews (PROSPERO) with the registration number CRD42023436966. Amendments made to the protocol were documented and justified accordingly.

### Search strategy

The databases PubMed and Embase (OVID) were searched for relevant studies using the following keywords: optimization model; malaria; control interventions; elimination interventions; and MeSH terms: resource allocation, models, linear programming, malaria, *Plasmodium*, communicable disease control, disease eradication. A detailed list of all search terms and results are available in Additional file 2. The search was run by the principal investigator. For each MeSH term and corresponding keyword, articles were sought by performing a title and abstract search on associated search terms. The results from the search of each MeSH term and corresponding keyword were combined exclusively using the Boolean operator ‘OR’. The final products of each keyword search were then combined using the Boolean operator ‘AND’ (Table 1). Records returned by the search were saved using the EndNote reference management software. Each record was screened by two independent reviewers using the Rayyan software. The screening process involved a review of the titles and abstracts of each record to identify potentially eligible records and exclude the records which were out of the scope of this review. The two reviewers then reviewed the full texts of the remaining records to identify eligible records for inclusion in the review. At the end of each stage, the reviewers discussed their findings to ensure uniformity and reviewed any discordances. A third reviewer was consulted in case of failure to resolve any discordances between the two reviewers. A list of all studies excluded at each stage of the screening process and the reasons for exclusion was made using the Rayyan software [31].

**Table 1:** Search strategy – models for malaria control optimization

#	Block	Search words
1	Optimization model	Resource allocat* OR allocative efficiency OR investment case OR dynamic model* OR programming OR dynamic programming OR dynamic analysis OR linear model* OR linear programming OR nonlinear model* OR nonlinear programming OR integer model* OR integer programming OR optimization AND model* OR optimization model* OR decision model* OR mathematical model* OR compartmental model* OR transmission model* OR agent-based model* OR individual based model*
2	Malaria	Malaria OR *Plasmodium*
3	Interventions	Control OR control interventions OR elimination OR pre-elimination OR elimination interventions OR eradication
4	#1 AND #2 AND #3	

### Inclusion criteria

Each record in the search were included if they met all of the following criteria: (1) Were scientific peer-reviewed journals written in English; (2) Were published before 30th June 2024; (3) Studies were included irrespective of the targeted geographical regions; (4) Studies were included irrespective of the population subgroups; (5) Studies containing an optimization model (mathematical or statistical); (6) All studies with human *Plasmodium* species; (7) Studies with two or more interventions; (8) Cost data were used in combination with the model outputs; (9) Outcomes/health benefits were clearly stated and/or measured.

### Exclusion criteria

The following records were excluded: (1) Editorials, commentaries, opinion papers, conference abstracts, media reports, letters, bulletins, pre-prints, grey literature; (2) Non-English language studies; (3) Systematic reviews, meta-analyses.

### Data extraction and synthesis

Data including (1) the first author and publication year; (2) geographic focus; (3) interventions used in analysis; (4) administrative level in the analysis; (5) populations considered; (6) time horizon of analysis; (7) method used to estimate health benefits (model structure); (8) *Plasmodium* species; (9) types of constraints; (10) data sources; (11) optimization goal; (12) epidemiological optimization, (13) cost optimization, (14) estimated time to elimination; (15) equity considerations in resource allocation; (16) conclusion of the article; were extracted from the selected studies into a Microsoft Excel Office 365 spreadsheet (Additional file 3). Missing or unclear data which were considered relevant triggered an email query to the corresponding authors of the respective studies and any additional information was included in the data extraction sheet if provided. All extracted data were double checked for errors by a second independent reviewer (SP) and discrepancies in entries were settled by appropriate discussion among both reviewers (RN and SP). A narrative synthesis of the study characteristics was performed.

### Quality assessment

The quality of all included studies was assessed using the joint International Society for Pharmacoeconomics and Outcomes Research-Society for Medical Decision Making Modelling Good Research Practices Task Force (ISPOR) [19]. The following criteria from ISPOR were used for quality assessment: conceptualising the model, dynamic transmission models, parameter estimation and uncertainty, and model transparency and validation (Additional file 4).

## Results

**Fig. 1:**
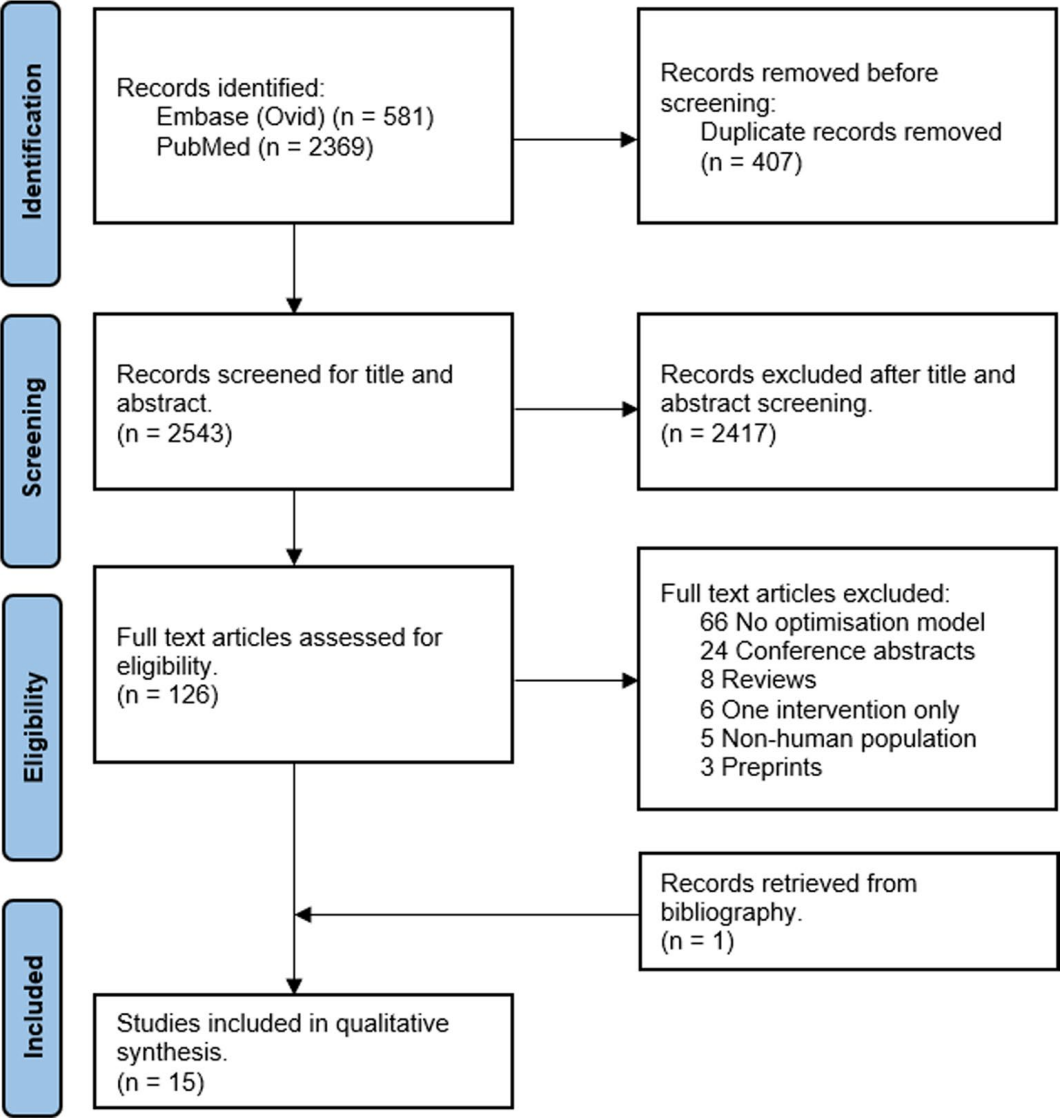
PRISMA diagram

A total of 2950 articles were identified from the search. When duplicates were removed, 2543 articles were screened for titles and abstracts, with 126 full-text articles assessed for eligibility. In total, 14 articles from the database search and 1 article from bibliography were included in the final analysis (Fig. 1).

The majority of modelling analyses (Table 2) were performed for countries in Africa [16–18, 25, 28, 32–34], with a single study (6.7%) spanning several countries in Africa and Asia [35]. Four studies (26.7%) focused solely on countries in Asia [10, 11, 29, 36], while two studies were done at the global scale [37, 38]. Myanmar and Thailand were the prominent Asian countries studied individually [10, 11, 36]. Among the African countries involved, two studies were from West Africa [16, 28], the others from East Africa [17, 34], and one from South Africa [32].

**Table 2:** Summary of models for malaria control optimization studies

Field	Frequency (%)	References
Total number of articles reviewed	15 (100)	[10, 11, 16–18, 25, 28, 29, 32–38]
Region of focus		
Africa	8 (53.3)	[16–18, 25, 28, 32–34]
Asia	4 (26.7)	[10, 11, 29, 36]
Africa and Asia	1 (6.7)	[35]
Global	2 (13.3)	[37, 38]
Number of interventions per article		
2	3 (20.0)	[10, 11, 17]
3	3 (20.0)	[34–36]
4	1 (6.7)	[33]
5	5 (33.3)	[18, 25, 29, 32, 38]
6 or more	3 (20.0)	[16, 28, 37]
*Plasmodium* species		
*P. falciparum*	13(86.7)	[10, 11, 16–18, 25, 28, 32–35, 37, 38]
*P. falciparum* + *P. vivax*	2 (13.3)	[29, 36]
Types of interventions		
ITNs/LLINs	14 (93.3)	[10, 11, 16–18, 25, 28, 29, 33–38]
IRS	12 (80.0)	[16–18, 28, 29, 32–38]
SMC	6 (33.3)	[16, 18, 25, 28, 33, 37]
Treatment	6 (33.3)	[25, 29, 35–38]
IPTp	5 (26.7)	[16, 25, 28, 37, 38]
MDA	3 (20.0)	[16, 18, 29]
Vaccine (RTS,S)	3 (20.0)	[25, 33, 38]
Surveillance	3 (20.0)	[29, 32, 37]
Community health workers	2 (13.3)	[10, 11]
IPTi (PMC)	2 (13.3)	[25, 38]
Social and behaviour change communication	2 (13.3)	[16, 28]
Larval source management	2 (13.3)	[16, 37]
Passive case detection	2 (13.3)	[28, 32]
2nd generation ITNs	1 (6.7)	[17]
Proactive case detection	1 (6.7)	[32]
Active case detection	1 (6.7)	[32]
Health system strengthening	1 (6.7)	[28]
Mass screen and treatment	1 (6.7)	[18]
Intermittent screen and treat	1 (6.7)	[34]
Population used in optimization		
General population	14 (93.3)	[10, 11, 16, 18, 25, 28, 29, 32–38]
Children under five years	1 (6.7)	[17]
Administrative level of data analysis		
National	13 (86.7)	[10, 11, 16–18, 25, 28, 29, 33, 35–38]
Subnational	9 (66.7)	[10, 11, 16, 18, 32, 34, 35, 37, 38]

All studies investigated WHO-recommended interventions for control or elimination targets (Table 2), such as insecticide-treated nets [10, 11, 16–18, 25, 28, 29, 33–38], seasonal malaria chemoprevention [16, 18, 25, 28, 33, 37], intermittent preventive treatment [16, 25, 28, 37, 38], indoor residual spraying [16–18, 28, 29, 32–38], mass drug administration [16, 18, 29], and diagnosis and treatment through community health workers or health facilities [10, 11, 25, 28, 29, 32, 35–38]. Other interventions investigated were surveillance [29, 32, 37], social and behaviour change communication [16, 28], larval source management [16, 37], active [32], passive [28, 32] and proactive case detection [32], health system strengthening [28], mass screen and treatment [18], and intermittent screen and treat [34]. Although not yet implemented in malaria-endemic countries at the time, the hypothetical implementation of the RTS,S vaccine was modelled in three studies, taking into account the epidemiological and cost components [25, 33, 38]. A full summary of all included articles can be found in Table 3.

**Fig. 2:**
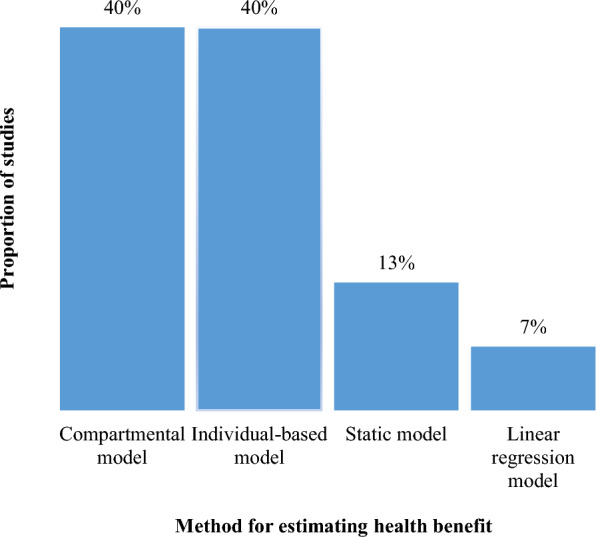
Method for estimating health benefits

Of the twelve studies that used dynamic transmission models in combination with economic/cost models for optimization, six (40.0%) used compartmental models [16, 17, 28, 29, 32, 38], and the other six (40.0%) used individual-based models [18, 25, 33–35, 37]. Two (13.0%) studies used static models [10, 11] and one (7.0%) study used a linear regression model for their analysis [36] (Fig. 2).

The administrative level of data analysis (Table 4) varied between national/multinational only (40.0%), national/multinational and subnational (46.7%), and subnational only (13.3%). A total of 9 (60.0%) studies used at least one source of country-specific epidemiologic or cost data or both for the transmission model parameterization, model calibration or economic analysis, while 6 (40.0%) studies used non-country-specific epidemiologic and cost data for the transmission model parameterization, model calibration or economic analysis. The country-specific data were sourced from district health information system (DHIS) databases, country level reports, NMCP reports, national malaria strategic plans, demographic and health survey (DHS) data, malaria indicator cluster survey (MICS) data, expert opinion and routine health system surveillance records [10, 11, 25, 28, 32, 34, 35, 37, 38]. The non-country-specific data were sourced from peer-reviewed literature, WHO reports, USAID reports, Global Fund reports, Malaria Atlas Project (MAP), procurement databases [16–18, 29, 33, 36] 

**Fig. 3:**
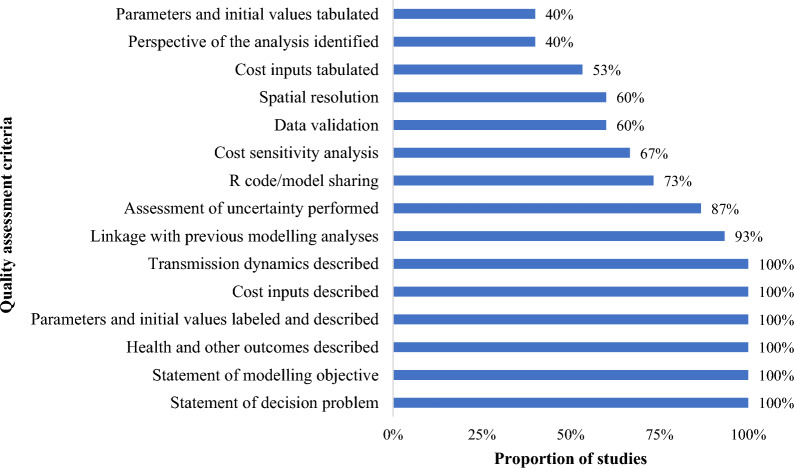
Quality assessment of included articles

Regarding the quality of the studies included in this review, all the studies included the statement of decision problem, the statement of modelling objective, describing health and other outcomes, labelling and describing parameters and initial values, describing cost inputs and transmission dynamics (Fig. 3). A few parameters that were not well explored by most studies were: the perspective of the analysis; and tabulating the parameters and cost inputs. Most studies shared the R code or model description either through an open-source platform or a previously published article.

**Table 3:** Summary of included articles

	Study identifier (Year of publication)	Geographic focus	Interventions or Scenarios	Administrative Level	Population	Time horizon of analysis (years)	Method of estimating health benefits	Species	Constraint	Data sources	Optimization goal	Optimization technique	Equity considerations in resource allocation
1	Sherrard-Smith et al. [17]	Tanzania, Uganda	Pyrethroid-only ITNs; Pyrethroid-PBO ITNs; IRS	National	Children under 5 years	3	Dynamic mathematical model (MINT tool); Scenario-based	*P. falciparum*	Minimum budget	Peer-reviewed literature (RCT)	Reducing annual cost per case averted	Stochastic programming	No
2	Njau et al. [32]	South Africa	Passive case detection; IRS; Active case detection; Proactive case detection; Border surveillance	Subnational (Province)	General population	11	Dynamic mathematical transmission model; Scenario-based	*P. falciparum*	Maximum budget	Local data (DHIS)	Achieve malaria elimination within a 10-year period	Stochastic programming	Yes, Malaria Surveillance Agents (MSAs)
3	Shretta et al. [28]	Ghana	Passive case detection; LLINs; IRS; Health system strengthening; Social and behavioural change; SMC; IPTp	National	General population	10	Dynamic compartmental transmission model; Scenario-based	*P. falciparum*	Minimum and maximum budget	Local data; World Malaria Reports; Peer-reviewed literature; Expert opinion	Provide economic evidence on risks of withdrawing financing as a strategy for resource mobilization	Metaheuristic method (Particle swarm optimization)	No
4	Shretta et al. [29]	Asia Pacific (22 countries)	LLINs; IRS; MDA; Treatment; Surveillance	Multi-national; National	General population	12	Dynamic compartmental transmission model; Scenario-based (METCAP)	*P. falciparum; P. vivax*	Minimum budget	World Malaria Reports; Peer-reviewed literature	Malaria elimination by 2030	Stochastic programming	No
5	Winskill et al. [25]	Sub-Saharan Africa	Treatment; LLINs; SMC; IPTi (PMC); RTS,S vaccine	Multi-national	General population	Not specified	Individual-based model	*P. falciparum*	Minimum and maximum budget	Peer-reviewed literature; Country level reports; WHO-CHOICE framework; Global Fund Price Reference Report	Maximise reduction in malaria transmission, case incidence and mortality with the least marginal cost	Non-linear programming	No
6	Sudathip et al. [36]	Thailand	Treatment; IRS; ITNs	National	General population	20	Two epidemiological models. Model A: Log-normal generalised linear regression model; Model B:; Scenario-based	*P. falciparum; P. vivax*	Minimum and maximum budget	Historical data; Expert opinion; Privately shared data	To measure the cost–benefit of a complete implementation of the NMES and thus assess the justification to invest in malaria elimination in Thailand	Linear programming	No
7	Drake et al. [10]	Myanmar	ITNs; CHWs	National; Subnational	General population	1	Geographically targeted resource allocation framework; Scenario-based	*P. falciparum*	Minimum budget	Local data; Reports	Using a geographic budget allocation network to maximise health benefits	Linear programming (knapsack)	Yes, Community Health Workers (CHWs)
8	Scott et al. [16]	Nigeria	LLINs; IRS; IPTp; SMC; Larval source management; MDA; Behavioural change communication	National; Subnational	General population	5	Geospatial epidemic (dynamic transmission) model; Optimization algorithm (Optima Malaria model); Scenario-based	*P. falciparum*	Maximum and minimum budget	Malaria Atlas Project (MAP); UN Population Division	Optimizing the allocation of scarce funding in targeted geographical regions to maximize reductions in malaria morbidity and mortality	Stochastic programming	No
9	Winskill et al. [33]	Sub-Saharan Africa	LLINs; IRS, SMC; RTS,S vaccine	Multi-national	General population	10	Individual-based model	*P. falciparum*	Cost-effectiveness threshold	Peer-reviewed literature; PMI, CHAI, MSF estimates	To derive the most cost-effective pathways for scaling-up malaria interventions in order to inform decisions about the introduction of the RTS,S malaria vaccine	Non-linear programming (gradient descent)	No
10	Winskill et al. [35])	Sub-Saharan Africa (19 countries); Greater Mekong Subregion	LLINs; IRS; ACTs	Multi-national; Subnational	General population	15	Individual-based model; Scenario-based	*P. falciparum*	Maximum and minimum budget	PMI reports; WHO World Malaria Reports; NMCPs; DHS; MICS; Peer-reviewed literature	To estimate the impact of PMI investments to date in reducing malaria burden and to explore the potential negative impact on malaria burden should a proposed 44% reduction in PMI funding occur	Linear programming	No
11	Patouillard et al. [37]	Global (All 97 malaria endemic countries)	All control interventions recommended by the WHO*	Multi-national; Subnational	General population	15	Individual-based model	*P. falciparum*	Maximum budget	World Malaria Reports; Global Rural–Urban Mapping Project; DHS; Procurement databases; Peer-reviewed literature; National malaria strategic plans; NMCP reports; WHO-CHOICE project; Key informant interviews	To estimate the financing required for malaria control and elimination over the 2016–2030 period	Stochastic programming	No
12	Walker et al. [18]	Sub-Saharan Africa	LLINs; IRS; SMC; MDA; Mass screen and treatment (MSAT)	Multi-national; Subnational; Pixel (Fine-scale)	General population	20	Individual-based model; Scenario-based	*P. falciparum*	Minimum budget	WHO Pesticide Evaluation Scheme (WHOPES); Peer-reviewed literature; PMI reports; Malaria Atlas Project (MAP)	To estimate the most cost-efficient strategies to achieve goals for reducing burden and transmission	Non-linear programming	No
13	Dudley et al. [38]	NA	LLINs; IRS; IPT; ACT; RTS,S vaccine	Multi-national; Subnational	General population	5	Integer linear program and compartment model; Scenario-based	*P. falciparum*	Maximum and minimum budget	Peer-reviewed literature; Country specific data; WHO Pesticide Evaluation Scheme (WHOPES)	Minimise person-days of malaria infection	Integer linear programming	No
14	Drake et al. [11]	Myanmar	ITNs; CHWs	National; Subnational	General population	1	Decision tree; Spatially explicit resource allocation model; Scenario-based	*P. falciparum*	Minimum budget	Three Millenium Development Goal (3MDG); Peer-reviewed literature; Routine health system surveillance records	To maximize impact from investment in ITN use and early diagnosis and treatment through malaria CHWs	Linear programming	Yes, Community Health Workers (CHWs)
15	Stuckey et al. [34]	Kenya	LLINs; IRS; Intermittent screen and treat (IST)	Subnational	General population	5	Microsimulation individual-based model (OpenMalaria); Scenario-based	*P. falciparum*	Cost-effectiveness threshold	Local survey data (MTC); WHO-CHOICE; Global Fund to Fight AIDS, Tuberculosis and Malaria Price and Quality Reporting Tool; Peer-reviewed literature	To address the cost effectiveness of feasible malaria control interventions	Stochastic programming	No

## Discussion

A total of 15 articles on models for malaria control optimization were identified from the literature. The majority of modelling analyses focused on countries in Africa and in the Asia Pacific regions. The interventions most commonly found in the analyses were ITNs, IRS, SMC and improved clinical case management. The data sources were country specific for some of the studies, although all studies had to rely on non-country-specific data to complete the analysis. The administrative level of analysis was at both the national and subnational levels, with a few studies having only subnational data analysis. There was a significant number of studies that had a budget constraint. However, very few carried out resource allocation within their constrained budgets. The studies included in this review exhibit various strengths and limitations, which will be outlined and examined below.

**Table 4:** Administrative level and data used in analysis

	Subgroup	Frequency (%)	References
Level of analysis	National/multinational only	6 (40.0%)	[17, 25, 28, 29, 33, 36]
	National/multinational and subnational	7 (46.7%)	[10, 11, 16, 18, 35, 37, 38]
	Subnational only	2 (13.3%)	[32, 34]
Data used in analysis	Country-specific data	9 (60.0%)	[10, 11, 25, 28, 32, 34, 35, 37, 38]
	Non-country-specific data	6 (40.0%)	[16–18, 29, 33, 36]

### Data quality and availability

While most studies used country-specific data for their analysis, they all had to complement their data sources with non-country-specific data for a more comprehensive analysis [10, 11, 25, 28, 32, 34, 35, 37, 38]. Moreso, there is an observed lower quality of the respective studies, as they did not meet all the criteria of the quality assessment such as not performing a cost sensitivity analysis and not defining the perspective for the analysis. There is a need for the accessibility of country-specific epidemiological and cost data, performing cost sensitivity analysis, and defining the perspective for the analysis in order to improve on the quality of the studies and render the results of these studies fit for purpose. There is limited evidence in peer-reviewed literature on modelling for malaria optimization in limited resource settings. The country focus of the modelling studies included in the review were not representative of the burden of malaria in sub-Saharan Africa. For instance, modelling analyses were performed in countries in East Africa [17, 34], West Africa [16, 28], and Southern Africa [32], with none performed in Central Africa. Multinational modelling analyses were performed in countries at risk of malaria in sub-Saharan Africa [18, 25, 33, 35]. However, there is a trade-off of doing these multinational analyses at scale, as subnational tailoring is needed for policy within NMCPs. In Asia, the two main countries that had modelling analyses performed were Myanmar [10, 11] and Thailand [36], while another study focused on 22 countries in the Asia Pacific for the modelling analyses [29]. The limited number of modelling analyses for malaria control optimization specific to countries within Africa and Asia is as a consequence of the lack or inaccessibility of country-specific epidemiologic and cost data at the national and subnational levels. This data gap limits future studies within the respective countries, limits the build-up of a critical mass of modellers within these countries, and makes it challenging for policymakers to make an evidence-based informed case to potential donors for future funding. There is a dire need to carry out more representative and country-specific modelling studies for resource allocation across Africa and the Asia Pacific, for malaria control or elimination.

### Model reproducibility and translational elements

There is a marked heterogeneity across all studies in the optimization modelling analyses used to. Specifically, as some studies use dynamic transmission models [16, 17, 28, 32], individual-based models [18, 25, 33–35, 37], or decision tree models [10, 11], the disparities in these optimization modelling analyses across studies make the comparison of the methods used to measure outcomes across these studies difficult. Also, the applications or software used in the development of these models for malaria control optimization analysis are varied [16, 17, 34], with limited knowledge or accessibility of the source code to the public. This limitation makes the reproducibility of the model across similar or neighbouring countries challenging and to some extent inaccessible. It is important to note that there are known current modelling efforts for informing allocation of malaria interventions in collaboration with country NMCPs [30, 39, 40]. These efforts on the use of non-optimization modelling techniques have gotten stakeholders involved in discussions surrounding the application of these models within countries, and in the development of policy engagement tools such as open access applications to facilitate the translation of these models [39–43].

### Interventions

All studies included vector control interventions such as the use of ITNs or IRS, most studies included improved clinical case management [10, 11, 18, 25, 28, 29, 34, 36–38], and some included surveillance [29, 32] in their analysis. The use of ITNs in combination with other prevention or treatment packages of interventions for optimal malaria control is usually recommended for use within the respective countries. Studies were identified that included a hypothetical implementation of the RTS, S vaccine in combination with standard interventions within the respective countries of interest [25, 33, 38]. There is a need for a consensus between countries to draw a clear path to malaria elimination, with country NMCPs driving the discussions around this consensus. There are key interventions that countries seeking elimination need to incorporate within their specific models for an eventual implementation. Some of these interventions are surveillance including active case detection and the implementation of the RTS,S vaccine. Accounting for these interventions would allow an analysis involving all possible intervention mixes, and provide more comprehensive outputs and outcomes, hence, a more realistic budget for the expected outcomes to lead to malaria elimination within the respective country.

### Equity considerations and subnational tailoring

For treatment interventions, only three studies considered targeting all high risk malaria populations including those that are hard-to-reach, by diagnosing and treating individuals in the most rural of communities with the help of community health workers [10, 11] or malaria surveillance agents [32]. With the most vulnerable or hard to reach populations falling within those at risk for malaria, the integration of community health workers within a community is a key aspect in maximising coverage [10, 11] for malaria control and elimination interventions within the population at risk. Also, the intervention of these community health workers is primordial in reducing mortality [44]. There is, therefore, the need to take into account equity considerations in the implementation of malaria interventions for impact. Finally, while three studies used resource allocation in their analyses [10, 11, 16], most studies used a health systems approach and did not tailor their analyses to subnational levels through the use of resource allocation techniques. National level analysis in the absence of subnational tailoring does not account for much heterogeneity in resource allocation, hence, inherently not providing optimal results. We recognise, however, that employing the same methodologies for a national level analysis across a large set of countries would be valuable as a means to equitably set budgets in a given endemic region.

Subnational allocation of resources provides a more specific attribution of the most effective interventions to the specific needs of each country or community, as can be seen in a recent study in Senegal [30]. Although there was no model used in the analysis, the authors present an aggregation of annual cost estimates of the intervention packages to provide data for better programmatic decision-making [30]. Overall, more efficient allocation of interventions within a country would mean dropping less efficacious interventions to focus more resources on other more relevant ones and thus ensure greater impact. Resource allocation is therefore invaluable in the establishment of informed and sustainable National Strategic Plans for endemic countries looking to control malaria. It is also a critical tool for the decision-making process in countries making a push towards elimination [32, 36], especially considering that interventions become more cost-ineffective as they near elimination.

## Limitations

This manuscript provides a systematic review of existing literature to identify models for malaria control optimization. However, the review has certain limitations, which are outlined below:

The review did not include abstracts from scientific conferences or other scientific meetings, potentially omitting important modelling studies conducted by national malaria control programs (NMCPs) to inform resource allocation decisions. And so, although the findings from this review shows that there are relatively few published modelling studies or examples that incorporate cost constraints and describe how to optimize limited budgets, the findings from this manuscript may not fully represent the actual use of models for malaria control optimization worldwide. The absence of these studies from the review limits the comprehensiveness of the findings, suggesting a need for the development of additional approaches to better capture and reflect the full range of modelling activities being undertaken globally.

The review specifically focused on models for malaria control optimization for resource allocation and their use. However, it did not account for non-optimization models that are also crucial for understanding the full spectrum of modelling tools available and their application in strategic planning for malaria control and resource allocation. Consequently, the review does not provide a complete picture of all modelling approaches that could be utilized by NMCPs for decision-making and planning.

These limitations suggest that while the review provides valuable insights into the current use of optimization models in malaria control, it may not fully capture the diversity of modelling efforts and their practical applications in real-world settings. Further research, including unpublished studies and non-optimization models, is necessary to obtain a more comprehensive understanding of the role of modelling in malaria control optimization.

## Conclusion

This review identified available optimization models for malaria resource allocation. The findings highlighted the need for country-specific modelling analysis for malaria control optimization, country-specific epidemiological and cost data for analysis, performing cost sensitivity analyses and defining the perspective for the analysis, with an emphasis on subnational tailoring for data collection and analysis for more accurate and good quality results. Such efforts should include all efficient prevention and treatment interventions, and surveillance and vaccination to inform context-specific control and elimination efforts respectively. It is critical that the future modelling efforts account for equity considerations and target at risk malaria populations that are hard-to-reach to maximize impact. Efforts towards developing publicly available applications of the models and sharing source codes to facilitate translation for policy engagement will enhance transparency, reproducibility and adaptability, and pave a way towards more harmonized models for malaria control optimization in the future.

## Supplementary Information

Additional File 1. PRISMA 2020 Checklist

Additional File 2. Search strategy and results from each database

Additional File 3. Complete data extraction of all studies included in final review

Additional File 4. Quality assessment of included articles

## Data Availability

The data that support the findings of this study were deposited into the Dataverse database and are available from the corresponding author upon reasonable request.

## Abbreviations

3MDG: Three Millenium Development Goal
CHAI: Clinton health access initiative
DHS: Demographic and health surveys
GTS: Global Technical Strategy
HBHI: High burden to high impact
HIV/AIDS: Human immunodeficiency virus/acquired immunodeficiency syndrome
IPTi: Intermittent preventive treatment in infants
IPTp: Intermittent preventive treatment in pregnancy
IRS: Indoor residual spraying
ISPOR: International society for pharmacoeconomics and outcomes research
ITN: Insecticide-treated bed nets
LLIN: Long-lasting insecticidal nets
LMIC: Low- and middle-income countries
MAP: Malaria atlas project
MDA: Mass drug administration
MeSH: Medical subject heading
MICS: Multiple indicator cluster surveys
MSF: Médecins Sans Frontières
NMCP: National malaria control program
PMC: Perennial malaria chemoprevention
PMI: President’s malaria initiative
PRISMA: Preferred reporting items for systematic reviews and meta-analysis
PROSPERO: Prospective register of systematic reviews
SMC: Seasonal malaria chemoprevention
UN: United Nations
USD: US Dollars
WHO: World Health Organization
WHO-CHOICE: World Health Organization Choosing Interventions that are Cost-Effective
WHOPES: WHO pesticide evaluation scheme
