# Supplementary material for: Models for malaria control optimization—a systematic review
Source: Malar J. 2024 Oct 3;23:295. doi: 10.1186/s12936-024-05118-3 (PMC11448400; doi:10.1186/s12936-024-05118-3)
Supplement: Supplementary file 2 — Additional File 2. Search strategy and results from each database [file 12936_2024_5118_MOESM2_ESM.docx]

**Additional file 2: Search strategy and results from each database**

1. Research question

Have existing optimisation models provided the desired outcomes for allocating resources for malaria interventions in resource-limited settings?

1. PICO format

Problem/Population – Populations of all ages at risk of having malaria in a resource-limited setting.

Intervention – Malaria prevention and case management interventions measured at endline.

Comparison – Malaria prevention and case management interventions measured at baseline.

Outcome – Health benefits (cases averted, deaths averted, incremental costs)

1. Search strategy PubMed
2. MeSH terms

| #1 Optimisation model | (("Resource Allocation"[Mesh]) OR "Models, Theoretical"[Mesh]) OR "Programming, Linear"[Mesh] |
| --- | --- |
| #2 Malaria | ("Malaria"[Mesh]) OR "Plasmodium"[Mesh] |
| #3 Interventions | ("Communicable Disease Control"[Mesh]) OR "Disease Eradication"[Mesh] |

1. tiab terms

| #1 Optimisation model | ((((((((((((((((((((((resource allocat*[Title/Abstract]) OR (allocative efficiency[Title/Abstract])) OR (investment case[Title/Abstract])) OR (dynamic model*[Title/Abstract])) OR (programming[Title/Abstract])) OR (dynamic programming[Title/Abstract])) OR (dynamic analysis[Title/Abstract])) OR (linear model*[Title/Abstract])) OR (linear programming[Title/Abstract])) OR (nonlinear model*[Title/Abstract])) OR (nonlinear programming[Title/Abstract])) OR (integer model*[Title/Abstract])) OR (integer programming[Title/Abstract])) OR (optimization[Title/Abstract] AND model*[Title/Abstract])) OR (optimization model*[Title/Abstract])) OR (optimisation[Title/Abstract] AND model*[Title/Abstract])) OR (optimisation model*[Title/Abstract])) OR (decision model*[Title/Abstract])) OR (mathematical model*[Title/Abstract])) OR (compartmental model*[Title/Abstract])) OR (transmission model[Title/Abstract])) OR (agent based model*[Title/Abstract])) OR (individual based model*[Title/Abstract]) |
| --- | --- |
| #2 Malaria | malaria[Title/Abstract] OR plasmodium[Title/Abstract] |
| #3 Interventions | (((((control[Title/Abstract]) OR (control interventions[Title/Abstract])) OR (elimination[Title/Abstract])) OR (pre-elimination[Title/Abstract])) OR (elimination interventions[Title/Abstract])) OR (eradication[Title/Abstract]) |

1. combining MeSH and tiab into search blocks

| #1 Optimisation model | (("Resource Allocation"[Mesh]) OR "Models, Theoretical"[Mesh]) OR "Programming, Linear"[Mesh] OR ((((((((((((((((((((((resource allocat*[Title/Abstract]) OR (allocative efficiency[Title/Abstract])) OR (investment case[Title/Abstract])) OR (dynamic model*[Title/Abstract])) OR (programming[Title/Abstract])) OR (dynamic programming[Title/Abstract])) OR (dynamic analysis[Title/Abstract])) OR (linear model*[Title/Abstract])) OR (linear programming[Title/Abstract])) OR (nonlinear model*[Title/Abstract])) OR (nonlinear programming[Title/Abstract])) OR (integer model*[Title/Abstract])) OR (integer programming[Title/Abstract])) OR (optimization[Title/Abstract] AND model*[Title/Abstract])) OR (optimization model*[Title/Abstract])) OR (optimisation[Title/Abstract] AND model*[Title/Abstract])) OR (optimisation model*[Title/Abstract])) OR (decision model*[Title/Abstract])) OR (mathematical model*[Title/Abstract])) OR (compartmental model*[Title/Abstract])) OR (transmission model[Title/Abstract])) OR (agent based model*[Title/Abstract])) OR (individual based model*[Title/Abstract]) |
| --- | --- |
| #2 Malaria | ("Malaria"[Mesh]) OR "Plasmodium"[Mesh] OR malaria[Title/Abstract] OR plasmodium[Title/Abstract] |
| #3 Interventions | ("Communicable Disease Control"[Mesh]) OR "Disease Eradication"[Mesh] OR (((((control[Title/Abstract]) OR (control interventions[Title/Abstract])) OR (elimination[Title/Abstract])) OR (pre-elimination[Title/Abstract])) OR (elimination interventions[Title/Abstract])) OR (eradication[Title/Abstract]) |
| #1 AND #2 AND #3 | **Number of hits: 2369** |

1. Search strategy Embase (Ovid)
2. resource allocation.ab,ti.
3. allocative efficiency.ab,ti.
4. investment case.ab,ti.
5. dynamic model.ab,ti.
6. programming.ab,ti.
7. dynamic programming.ab,ti.
8. dynamic analysis.ab,ti.
9. linear model.ab,ti.
10. linear programming.ab,ti.
11. nonlinear model.ab,ti.
12. nonlinear programming.ab,ti.
13. integer model.ab,ti.
14. integer programming.ab,ti.
15. optimi$ation model.ab,ti.
16. decision model.ab,ti.
17. mathematical model.ab,ti.
18. compartmental model.ab,ti.
19. transmission model.ab,ti.
20. agent based model.ab,ti.
21. individual based model.ab,ti.
22. 1 or 2 or 3 or 4 or 5 or 6 or 7 or 8 or 9 or 10 or 11 or 12 or 13 or 14 or 15 or 16 or 17 or 18 or 19 or 20
23. malaria.ab,ti.
24. plasmodium.ab,ti.
25. 22 or 23
26. 21 and 24
27. control.ab,ti.
28. control interventions.ab,ti.
29. elimination.ab,ti.
30. pre-elimination.ab,ti.
31. elimination interventions.ab,ti.
32. eradication.ab,ti.
33. 26 or 27 or 28 or 29 or 30 or 31
34. 25 and 32

**Number of hits: 581**

1. Total number of hits

PubMed = 2369

Embase (Ovid)= 581

**Total = 2950**

Duplicates removed = 407

Records screened for title and abstract (after deduplicates) = 2543

Records excluded after title and abstract screening = 2417

Reasons for exclusion

- No models in study = 1260
- No cost component = 312
- Animal population = 295
- Other disease = 268
- No intervention/scenario = 108
- Different outcome = 54
- Co-infections = 43
- Review = 42
- Reports = 23
- Cost/cost-effectiveness = 12

Full text articles assessed for eligibility = 126

Records excluded (after reading full articles) = 112

Reasons for exclusion

- No optimisation model = 66
- Conference abstract = 24
- Review = 8
- One intervention only = 6
- Non human population = 5
- Preprint = 3

Records retrieved from bibliography = 1

Records retained for qualitative analysis = 15
