# Supplementary material for: Models for malaria control optimization—a systematic review
Source: Malar J. 2024 Oct 3;23:295. doi: 10.1186/s12936-024-05118-3 (PMC11448400; doi:10.1186/s12936-024-05118-3)
Supplement: Supplementary file 4 — Additional File 4. Quality assessment of included articles [file 12936_2024_5118_MOESM4_ESM.docx]

**Additional File 4: Quality assessment of included articles**

| **Study identifier (year of publication)** | **Statement of decision problem** | **Statement of modeling objective** | **Perspective of the analysis identified** | **Health and other outcomes described** | **R code/model sharing** | **Parameters and initial values tabulated** | **Parameters and initial values labeled and described** | **Cost inputs described** | **Cost inputs tabulated** | **Transmission dynamics (e.g., incidence, prevalence) described** | **Assessment of uncertainty performed** | **Linkage with previous modeling analyses** | **Sensitivity analysis** | | **Data validation** | **Spatial resolution** |
| --- | --- | --- | --- | --- | --- | --- | --- | --- | --- | --- | --- | --- | --- | --- | --- | --- |
|  |  |  |  |  |  |  |  |  |  |  |  |  | **Yes or No** | **Type** |  |  |
| **Sherrard-Smith et al (2022)^11^** | Yes | Yes | No | Yes | No | No | Yes | Yes | No | Yes | Yes | No | No | NA | No | No |
| **Njau et al (2021)^21^** | Yes | Yes | No | Yes | Yes | No | Yes | Yes | No | Yes | No | Yes | No | NA | Yes | Yes |
| **Shretta et al (2020)^13^** | Yes | Yes | Yes | Yes | Yes | No | Yes | Yes | No | Yes | Yes | Yes | Yes | Stochastic | Yes | No |
| **Shretta et al (2019)^14^** | Yes | Yes | No | Yes | Yes | No | Yes | Yes | No | Yes | Yes | Yes | Yes | Stochastic | No | No |
| **Winskill et al (2019)^9^** | Yes | Yes | Yes | Yes | Yes | No | Yes | Yes | Yes | Yes | Yes | Yes | No | NA | Yes | No |
| **Sudathip et al (2019)^22^** | Yes | Yes | Yes | Yes | No | No | Yes | Yes | Yes | Yes | No | Yes | No | NA | No | No |
| **Drake et al (2017)^16^** | Yes | Yes | No | Yes | No | No | Yes | Yes | No | Yes | Yes | Yes | No | NA | Yes | Yes |
| **Scott et al (2017)^12^** | Yes | Yes | No | Yes | Yes | Yes | Yes | Yes | Yes | Yes | Yes | Yes | Yes | Multivariate | No | Yes |
| **Winskill et al (2017a)^23^** | Yes | Yes | No | Yes | Yes | No | Yes | Yes | No | Yes | Yes | Yes | Yes | Multivariate | No | No |
| **Winskill et al (2017b)^24^** | Yes | Yes | No | Yes | Yes | Yes | Yes | Yes | No | Yes | Yes | Yes | Yes | Multivariate | Yes | Yes |
| **Patouillard et al (2017)^25^** | Yes | Yes | Yes | Yes | No | Yes | Yes | Yes | Yes | Yes | Yes | Yes | Yes | Probabilistic | Yes | Yes |
| **Walker et al (2016)^26^** | Yes | Yes | No | Yes | Yes | No | Yes | Yes | Yes | Yes | Yes | Yes | Yes | Not specified | No | Yes |
| **Dudley et al (2016)^27^** | Yes | Yes | No | Yes | Yes | Yes | Yes | Yes | Yes | Yes | Yes | Yes | Yes | Scenario | Yes | Yes |
| **Drake et al (2015)^28^** | Yes | Yes | Yes | Yes | Yes | Yes | Yes | Yes | Yes | Yes | Yes | Yes | Yes | Univariate | Yes | Yes |
| **Stuckey et al (2014)^29^** | Yes | Yes | Yes | Yes | Yes | Yes | Yes | Yes | Yes | Yes | Yes | Yes | Yes | Univariate | Yes | Yes |

NA, not applicable

**Quality scores of included articles**

**Selected best practices for optimization modeling from the joint ISPOR-SMDM task force**

| Recommendation number | Description |
| --- | --- |
| II-2 | A clear, written statement of the decision problem, modeling objective, and scope of the model should be developed. This should include: the spectrum of disease considered, perspective of the analysis, target population, alternative interventions, health and other outcomes, and time horizon. |
| II-4 | The conceptual representation of the decision problem should be used to identify key uncertainties in model structure where sensitivity analyses could inform the impact of structural choices. For example, where a lifetime horizon is used, the impact of alternative methods of extrapolating beyond the observed data should be explored. |
| VI-1 | The systematic examination and reporting of uncertainty are hallmarks of good modeling practice. All modeling studies should therefore include an assessment of uncertainty as it pertains to the decision problem being addressed. |
| VI-3 | Terminology to describe concepts relating to parameter estimation and representation of uncertainty varies within the medical decision modeling field and in comparison to related fields. Authors should be aware of this and seek to carefully define their use of terminology to avoid potential confusion. |
| VI-4 | All decision models will have parameters that need to be estimated. In populating models with parameter estimates, analysts should conform to the broad principles of evidence based medicine. For example, analysts should: seek to identify and incorporate all relevant evidence, rather than cherry picking the best single source of evidence for that parameter; use best practice methods to avoid potential biases in parameter estimates that might arise (for example, when estimating treatment effectiveness from observational sources); and employ formal evidence syntheses techniques (meta-analysis and network meta-analysis) as appropriate. |
| VII-1 | Every model should have non-technical documentation that is freely accessible to any interested reader. At a minimum it should describe in non-technical terms the type ofmodel and intended applications; funding sources; structure of the model; inputs, outputs, other components that determine the model’s function, and their relationships; data sources; validation methods and results; and limitations. |
| VII-2 | Every model should have technical documentation, written in sufficient detail to enable a reader with the necessary expertise to evaluate the model and potentially reproduce it. The technical documentation should be made available openly or under agreements that protect intellectual property, at the discretion of the modelers. |
| VII-3 | Validation of a model should include an evaluation of face validity of the structure, evidence, problem formulation, and results of the model. A description of the process used to evaluate face validity should be made available on request. Evaluation of face validity should be made by people who have expertise in the problem area, but are impartial to the results of an analysis. If face validation raises questions about a model, these issues should be discussed by the modelers in their report of an analysis. |
| VII-5 | Modelers should search for previously published modeling analyses of the same or similar problems and discuss insights gained from similarities and differences in results. |
